# Critical analysis of digitalis glycosides: declining use but increasing poison-center exposure cases for digitoxin

**DOI:** 10.1007/s00228-026-04099-3

**Published:** 2026-06-23

**Authors:** Paul Antek Matthias Ecker, Andreas Schaper, Hans Jörg Bräunig, Roland Seifert

**Affiliations:** 1 Institute of Pharmacology, Hannover Medical School, Hannover, D-30625 Germany; 2 GIZ-Nord Poisons Centre for Bremen, Hamburg, Niedersachsen and Schleswig-Holstein Germany; 3 Georg-August-University of Göttingen, Göttingen, D-37075 Germany

**Keywords:** Cardiovascular drug prescription, Na^+/^K^+^-ATPase inhibitors, Na^+/^K^+^-ATPase inhibitor intoxications, Pharmacoeconomics, Evidence-based medical practice, Germany

## Abstract

**Purpose & Background:**

Belonging to one of the oldest drug classes in cardiovascular-medicine, the scientific interest in Na^+/^K^+^-ATPase inhibitors (NKA-inhibitors, digitalis glycosides) has increased once again after the publication of the recent DIGIT-HF study. This analysis and scoping review aim to assess long-term prescribing trends of NKA-inhibitors and poison center data to investigate the discrepancy between declining use and increasing poison center reports involving digitoxin in Germany.

**Methods:**

By using prescription data (defined daily dose, DDD) and poison center records between 1990 and 2023 in Germany we conducted a pharmacological, pharmacoeconomic and toxicological analysis of the four NKA-inhibitors digitoxin, digoxin, acetyldigoxin and metildigoxin. For identifying trend changes and prescribing trends, we used joinpoint regression and DDD-half-time analyses.

**Results:**

The DDD of NKA-inhibitors declined substantially over our investigated timeframe. Reported exposure cases involving digitoxin, recorded by the GIZ-Nord poison center, showed a significant and continuous increase. Population-adjusted exposure cases increased by 4.65% per year. Exposure-adjusted exposure cases per 1 mio. DDD increased by 11.80% per year. The data for reported cases involving digoxin showed large year-to-year variability and no significant increase in reported case numbers. The PRISM DDD-half-time analysis showed big differences between the four substances, with digitoxin exhibiting the longest DDD-half-time despite the absence of supportive evidence in the form of clinical trials, suggesting a dissociation between evidence and real-world prescriptions.

**Conclusions:**

Prescription volumes decrease but digitoxin is associated with steadily increasing reports of exposure cases from poison center data. This indicates discrepancies between prescribing trends and real-world exposure patterns and highlights the importance for patient selection and careful monitoring, especially in elderly patients.

**Supplementary Information:**

The online version contains supplementary material available at 10.1007/s00228-026-04099-3.

## Introduction

Na^+/^K^+^-ATPase inhibitors (NKA-inhibitors, digitalis glycosides) are an old-established substance group for the treatment of heart failure and atrial fibrillation [1–3]. Despite the availability of modern alternatives they are still being used in today’s clinical practice, particularly in vulnerable patient groups [3, 4]. However, the evidence for their efficacy and safety is still limited [4]. Most data originates from few historical trials like the DIG (1997) which demonstrated a reduction in hospitalizations for heart-failure but showed no effect for the reduction of overall mortality [3]. Modern real-world evidence especially concerning the toxicity of NKA-inhibitors together with prescription numbers is sparse. The narrow therapeutic window, the complex pharmacokinetics as well as the potential for drug interactions contributes to the controversy of their ongoing clinical use. Recently the scientific interest in this drug category has been reignited, particularly following the publication of DIGIT-HF, the first clinical trial investigating digitoxin as primary substance, suggesting a potential benefit as add-on therapy in selected patients with HFrEF [5]. Digitoxin still is the main NKA-inhibitor used in Germany, despite a long period with limited clinical evidence. However, the relationship between prescribing behavior and reported exposure rates derived from poison center data in a real-world setting has not been investigated yet. This evidence-gap is especially relevant in the context of evolving evidence and treatment strategies. This study therefore aims to critically evaluate the pharmacological, pharmacoeconomic and toxicological development of digitalis use in Germany from 1990 to 2023 and to relate these trends to key influencing factors and reported exposure cases based on population and exposure-adjusted real-world data.

## Materials and methods

The study was conducted as scoping review with an original analysis of DDD and intoxication datasets. The review component is intended to contextualize observed trends in DDD and reported exposure cases of the GIZ-Nord concerning NKA-inhibitors. PRISMA ScR guidelines were used as a reporting guideline for ensuring transparency for this exploratory scoping review, without implying to conduct full systematic research. Detailed page allocation according the PRISMA-ScR criteria is displayed in Table S1. There was no review protocol registered before performing this analysis. The DDD and gross revenue numbers were obtained from the scientific institute of the AOK (WIdO). By using the official population numbers from the Federal Statistical Office of Germany (Statistisches Bundesamt (Destatis), Genesis-Online; data-license by-2-0) DDD-values were standardized into DDD/100-inhabitants/day. To put the received data into perspective, they were analyzed in tabular form and graphically visualized with Excel. The individual curves and data trends were then further investigated conducting a focused screening review of studies on the following medical and scientific databases:

https://pubmed.ncbi.nlm.nih.gov

https://clinicaltrials.gov

**Fig. 1:**
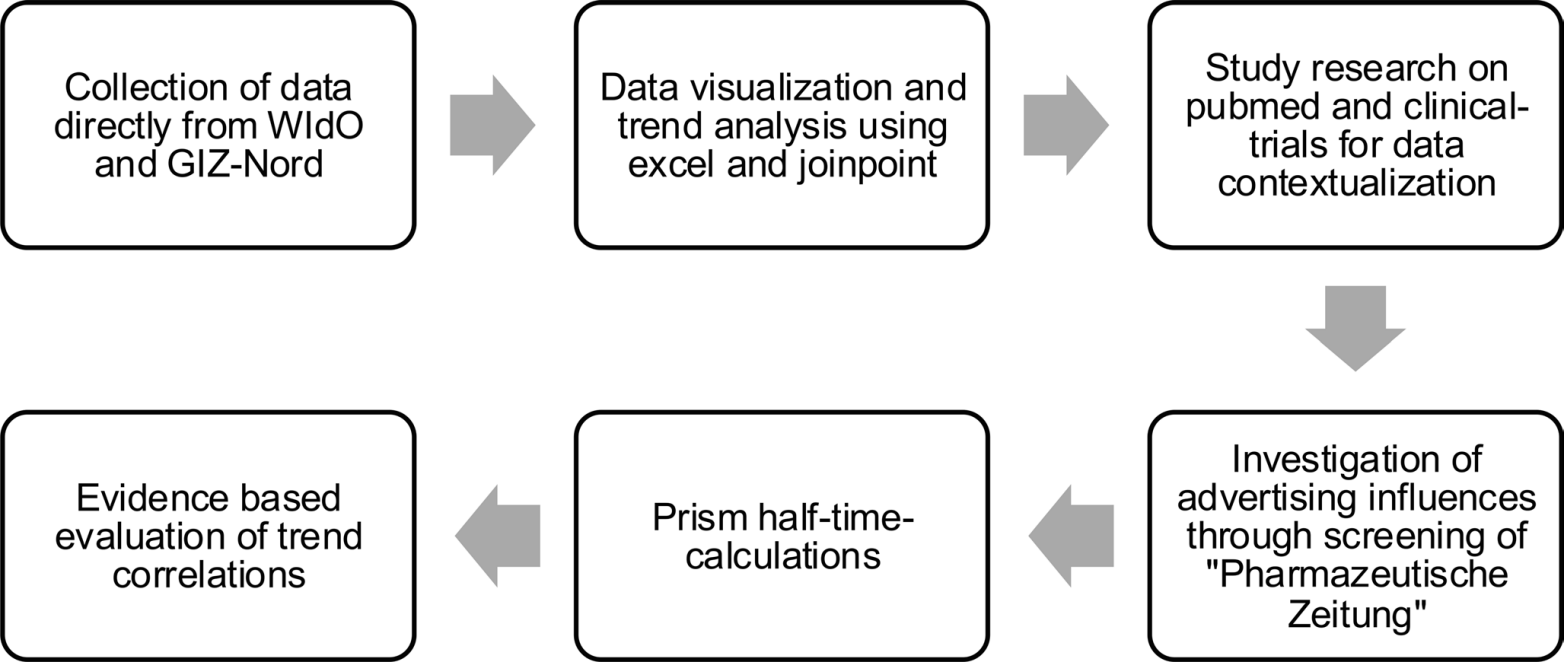
Schematic overview of study design and workflow for the evaluation of prescribing trends and poison-center reported exposure cases of NKA-inhibitors

Clinical Trials was used to identify studies investgating the NKA-inhibitors digoxin, digitoxin, acetyldigoxin and metildigoxin to receive a study count. Each substance was researched individually on ClinicalTrials.gov by entering the substances in the ‚intervention‘ field. No additional filters were used. The majority of the research on ClinicalTrials was conducted in 2025, last search January 20, 2026. A total of 42 studies were found for digoxin, 1 study was found for digitoxin, 0 studies were found for acetyldigoxin and metildigoxin. All studies identified on ClinicalTrials were manually screened. Studies were included when they investigated a cardiac indication and the NKA-inhibitor was an active intervention or comparator. Studies without cardiac indication or NKA-inhibitors as background therapy or relevance to objectives of this review were excluded. Studies that matched this criteria were documented in a structured table including NCT-ID, indication, intervention, study population and availability of results. Additionally pub-med was searched to identify NKA-inhibitor studies within the investigated time-period 1990–2023. No additional filters were used, the results were manually screened to identify the most relevant studies investigating the cardiac indications of NKA-inhibitors. Our focus was to identify major and influencial studies in the investigated timeframe to contextualize the identified DDD-trends. Major studies were displayed and categorized in a study-traffic light to visually illustrate the methodological characteristics, including study design, randomization, masking, presence or absence of a placebo group as well as primary outcome. Green indicates the presence of the factor, red the absence and yellow that it was partially met or contains limitations (e.g. observational design or post-hoc analyses). The traffic-light is intended to support the readers interpretation and does not represent a formal study quality assessment. A formal critical assessment or risk of bias assessment was not performed. This study intentionally does not claim to conduct a full systematic review to maintain the focus of this paper as an original data analysis. The influence of study results was studied in connection with DDD numbers. To investigate whether the identified studies and influence factors had a significant impact on trend development, we used the Joinpoint Regression Program (Version 5.4.0, National Cancer Institute USA). For the identification of major trends and turning-points, we used the grid-search-method with automatic model-selection based on the Weighted Bayesian Information Criterion (WBIC). Additionally, we calculated separate pre- and post-event linear models, restricted to zero joinpoints each, to receive comparable results. The results are presented as Annual Percent Change (APC) with 95% confidence intervals (CI). For exploration of potential association between certain events and DDD-development we conducted pre- and post-event joinpoint analyses. These analyses were used to estimate APCs and to allow descriptive comparison of trend development but do not formally test differences between the periods. To identify statistically significant changes between periods, we used the joinpoint regression model. For both whole-joinpoint analysis and pre/post-analyses we used the uncorrelated constant-variance error model. Because at least four data points after a potential joinpoint are required for a valid trend assessment, events after 2018 couldn’t be evaluated. According the joinpoint- analyses based on GIZ-Nord data and population data, we conducted population adjusted analyses of NKA-inhibitor exposure cases per 100.000 inhabitants. Based on the Destatis data from the four GIZ-Nord federal states, we adjusted the DDD-data received by the WidO according to the percentage of the catchment area and performed joinpoint analyses for exposure cases per 1mio. DDD to receive exposure adjusted case rates for digitoxin and digoxin. For the GIZ-Nord data analyses we used the uncorrelated constant variance error model and permutation test for model selection. To verify the sensitivity of our model we repeated our calculations with poisson-variance (tables S14-S17 supplement). For years where zero exposure cases were reported, (digoxin 2017), a small constant (0.5) was used to allow log-transformation within the regression model. We chose this approach to avoid instability and artificial distortion when choosing values near to zero. Studies with results, found on clinical trials were further included into a DDD-half-time calculation using Prism to assess the extent to which the prescribing practice of NKA-inhibitors is based on evidence. DDD-half-time is a calculated value representing the amount of time where the defined daily dosages reach half of their initial volume. The DDD-half-time therefore is an important device to quantify the DDD-reduction over the investigated timeframe and to link the reduction to possible trigger factors. A short DDD-half-time was regarded as an indicator for a low evidence level and subsequent clinical practice, whereas a long DDD-half-time was regarded as an indicator for a high level of evidence and subsequent clinical practice. To be considered relevant for this calculation the study needed to be a multicentric RCT with a sizeable study population within the investigated timespan (1990–2023). Withdrawal studies were not included into the study review. Considering the question whether advertising influences may have had an effect on the prescription of the analyzed substances, the medical journal “Pharmazeutische Zeitung” was analyzed for advertisements from 1992 to 1995. Medical textbooks were additionally analyzed to observe a change in individual indications. Reported exposure rates for the NKA-inhibitors were directly received from the German Poison Information Centre North (GIZ-Nord) and display all reported exposure cases from the four northern German federal states (Bremen, Hamburg, Niedersachsen, Schleswig-Holstein) Fig. 1.

## Results

### Study analysis

**Fig. 2:**
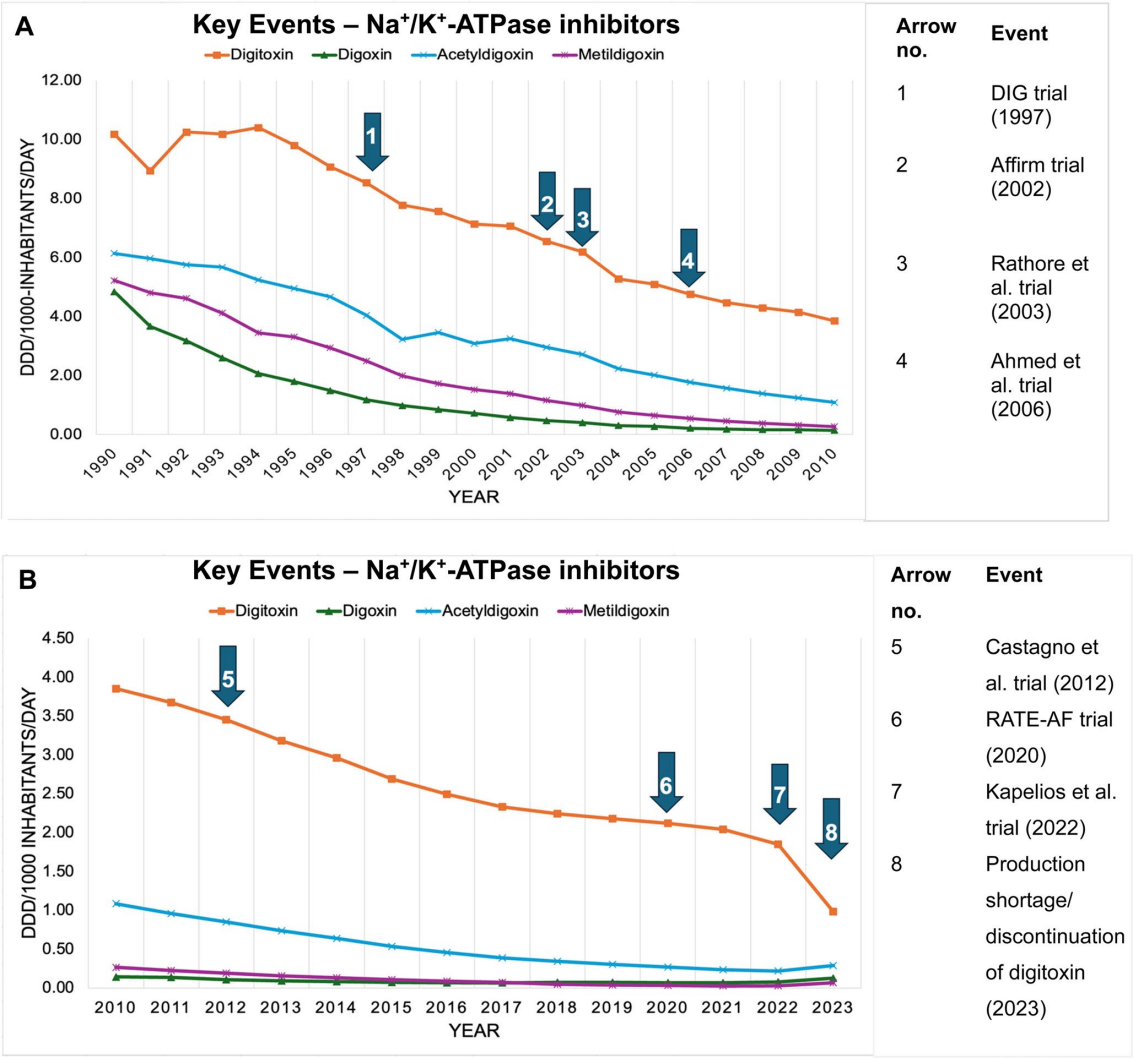
**A** Key events for NKA-inhibitors within the investigated timespan. According key-event legend on the right. (Data source: WidO). **B** Key events for NKA-inhibitors within the investigated timespan. According key-event legend on the right. (Data source: WidO)

**Fig. 3:**
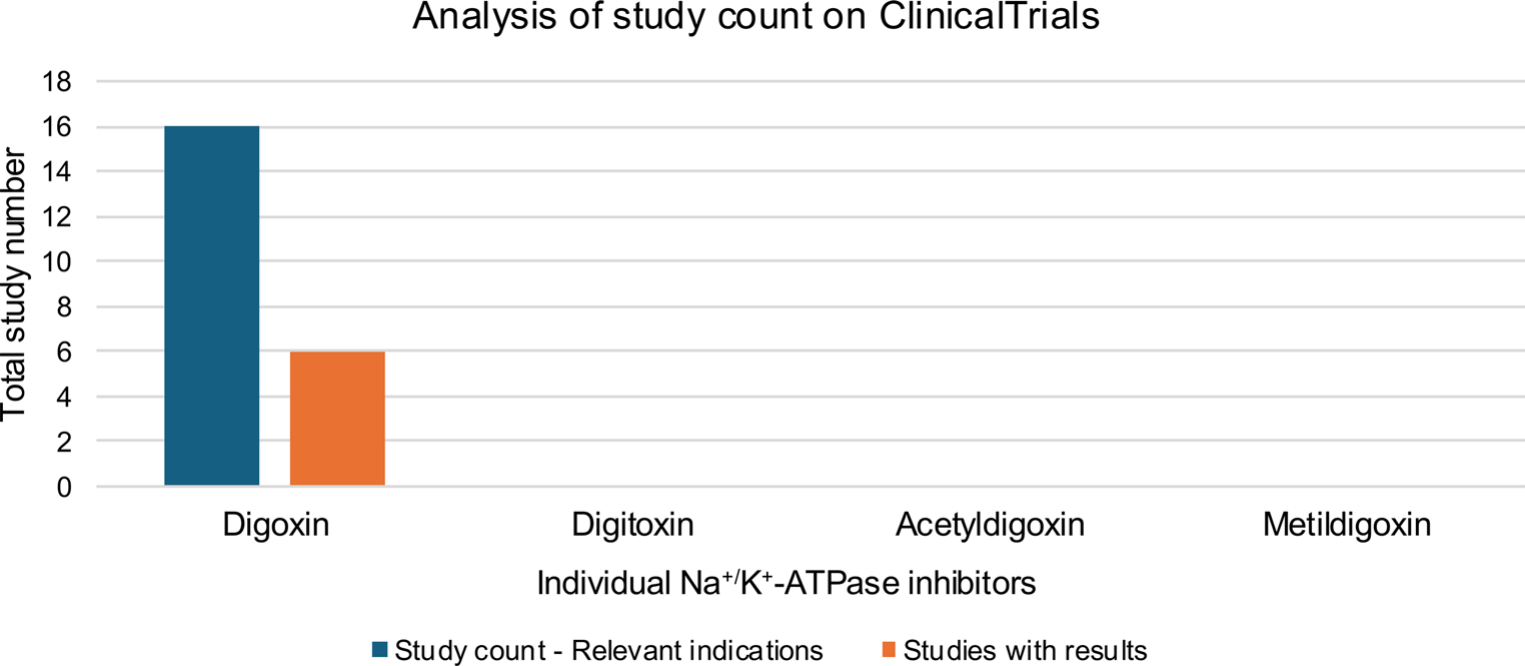
Search results of total study count of NKA-inhibitors on ClinicalTrials displayed in a column chart. blue: total studies with indications relevant to this analysis found for the individual NKA-inhibitor, orange: total studies with results available. DIGIT-HF was registered on EudraCT and is therefore not represented in our ClinicalTrials.gov study count

Figure 2 shows the time course of prescribed DDD of the various NKA-inhibitors in Germany from 1990 to 2023. Figure 3 shows that all clinical trials registered on ClinicalTrials focused on digoxin. We identified eight NKA-inhibitor-trials within the investigated timespan. The study analysis found one major clinical study investigating the efficacy and prognostic treatment effect (The Digitalis Investigation Group – DIG 1997). Three post-hoc analyses of the DIG study were performed. The DIG (1997) represents the only randomized controlled trial (RCT) that was conducted with a masked design and a placebo group (Table 1). Although the DIG-trial showed a reduction in hospitalizations, it failed to prove a significant mortality reduction for digoxin [3]. Figure 2A and B, indicate that this finding only had minimal impact on the clinical use of this substance. Subsequent post-hoc analyses showed that digoxin may be able to reduce mortality in patients with heart failure and reduced ejection fraction (HFrEF) when administered in the correct dosage [6, 7]. Nevertheless, this effect has to be verified in a clinical RCT. In addition to the DIG, there were two other clinical RCTs that investigated digoxin: AFFIRM (2002) and RATE-AF (2020).

**Table 1:**
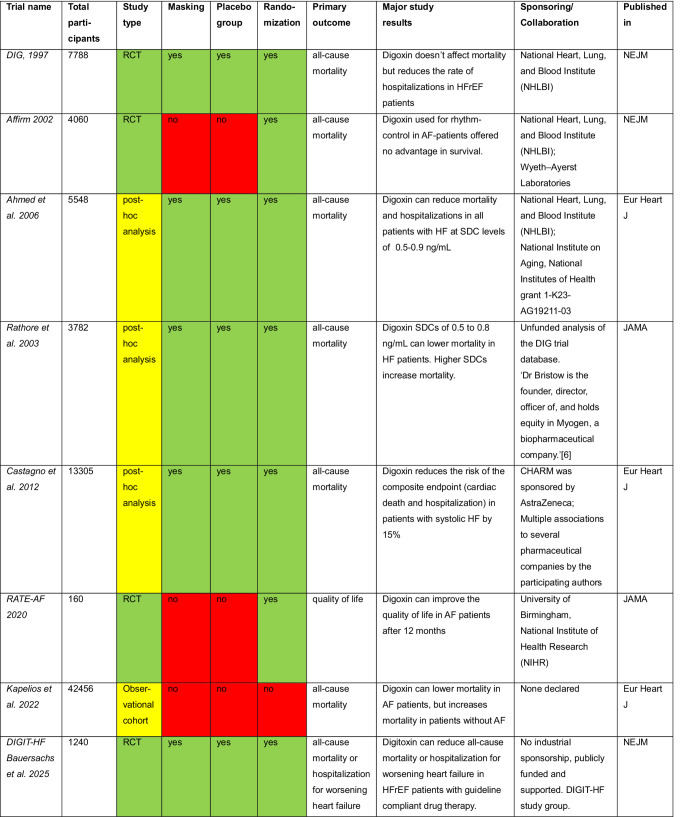
Overview of selected studies on pub-med presented in a table. Features color-coded: Green, full presence of more robust methodological factors (e.g. RCT design). Yellow, partially present factors or presence of limitations (e.g. post hoc analyses or non-randomized) Red, methodological factors that are absent

However, these two studies showed deficiencies in placebo- and masking aspects [8, 9]. Although, the RATE-AF study had a relatively small population size, with only 160 participants [9]. Looking ahead, the multicenter, randomized, double-blind, placebo-controlled DIGIT-HF trial had the objective to evaluate the clinical evidence of digitoxin in patients with chronic HFrEF in addition to guideline-based therapy [10]. The primary composite endpoint was all-cause mortality or hospitalization for heart failure [10]. The DIGIT-HF trial identified a significant reduction in the composite endpoint for certain digitoxin plasma concentrations for patients with chronic HFrEF [5]. The DIGIT-HF trial currently is the only clinical trial for the investigation of digitoxin as the primary substance and the only trial providing scientific evidence for the use of digitoxin in clinical practice. In general, there seems to be a clear predominance of studies investigating digoxin, compared to digitoxin (Fig. 3). Until publication of the DIGIT-HF trial, most of the scientific evidence relied on the results of the DIG trial in 1997, which investigated digoxin as primary substance. Since the DIGIT-HF trial was registered on EudraCT, it was therefore not included in our study count on ClinicalTrials.gov in Fig. 3. In 2023 Merck, the main producer of digitoxin drugs permanently discontinued the production of Digimerck^®^ due to economic reasons and a production material shortage (Bauersachs et al., 2023; Gelbe Liste, 2022). This led to significant DDD-reduction in all substances after 2021 (Figs. 2B).

### Joinpoint analysis – results

**Fig. 4:**
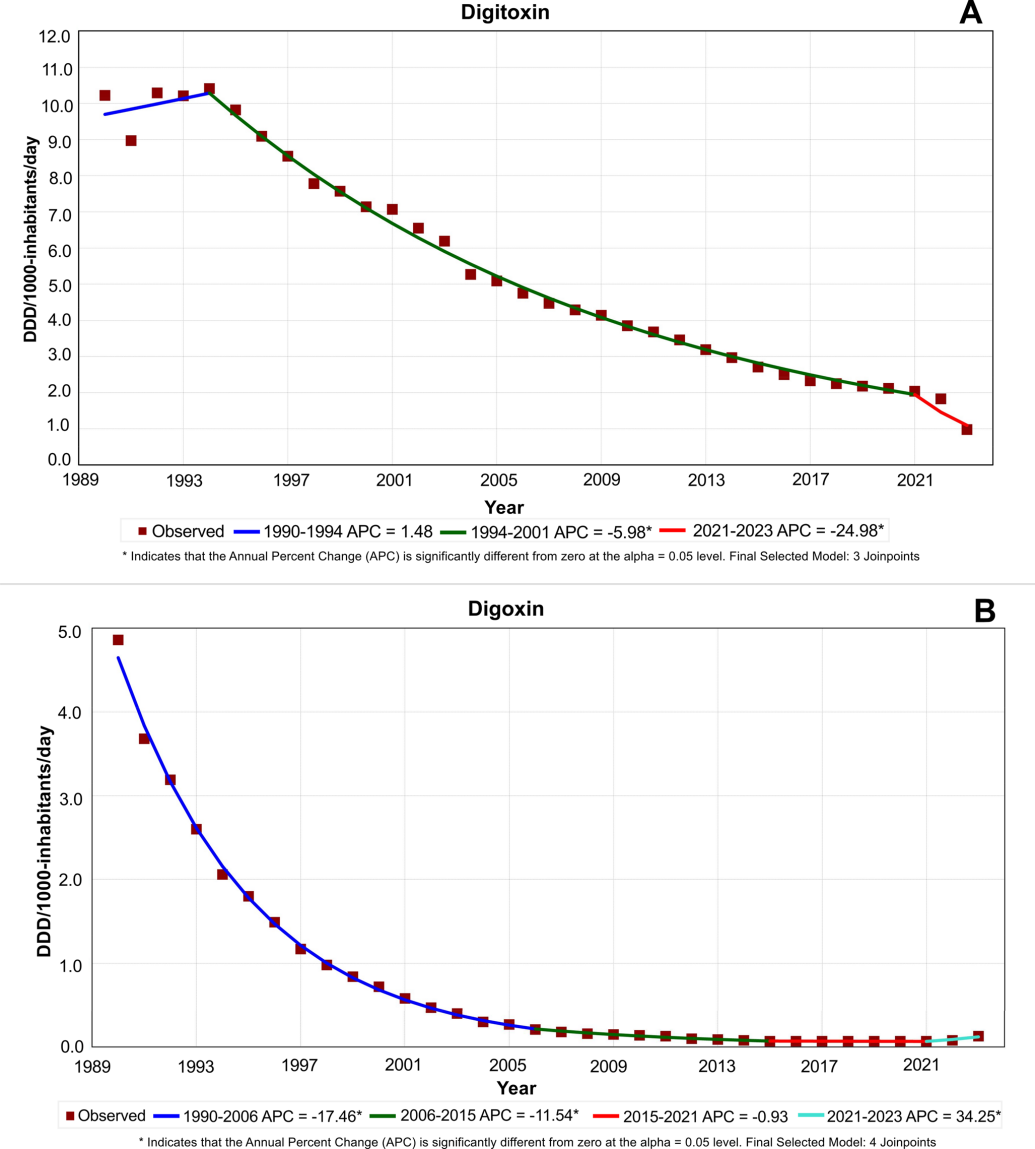
Multi-panel display of joinpoint analyses for digitoxin and digoxin use (DDD/1000-inhabitants/day) from 1990–2023. (**A**) Digitoxin; (**B**) Digoxin. Brown square points display the DDD/1000-inhabitants/day. The coloured lines represent the automatically recognized trend segments. A different colour represents a different trend segment. Detailed data tables according the DDD/1000-inhabitants/day for the four NKA-inhibitors (APC, Upper- and lower CI, P-values) are displayed in Tables 2–5 in the supplementary material. Y-axis was adjusted individually for each substance to improve visualization of substance specific trends. Comparison should focus on relative trends

**Fig. 5:**
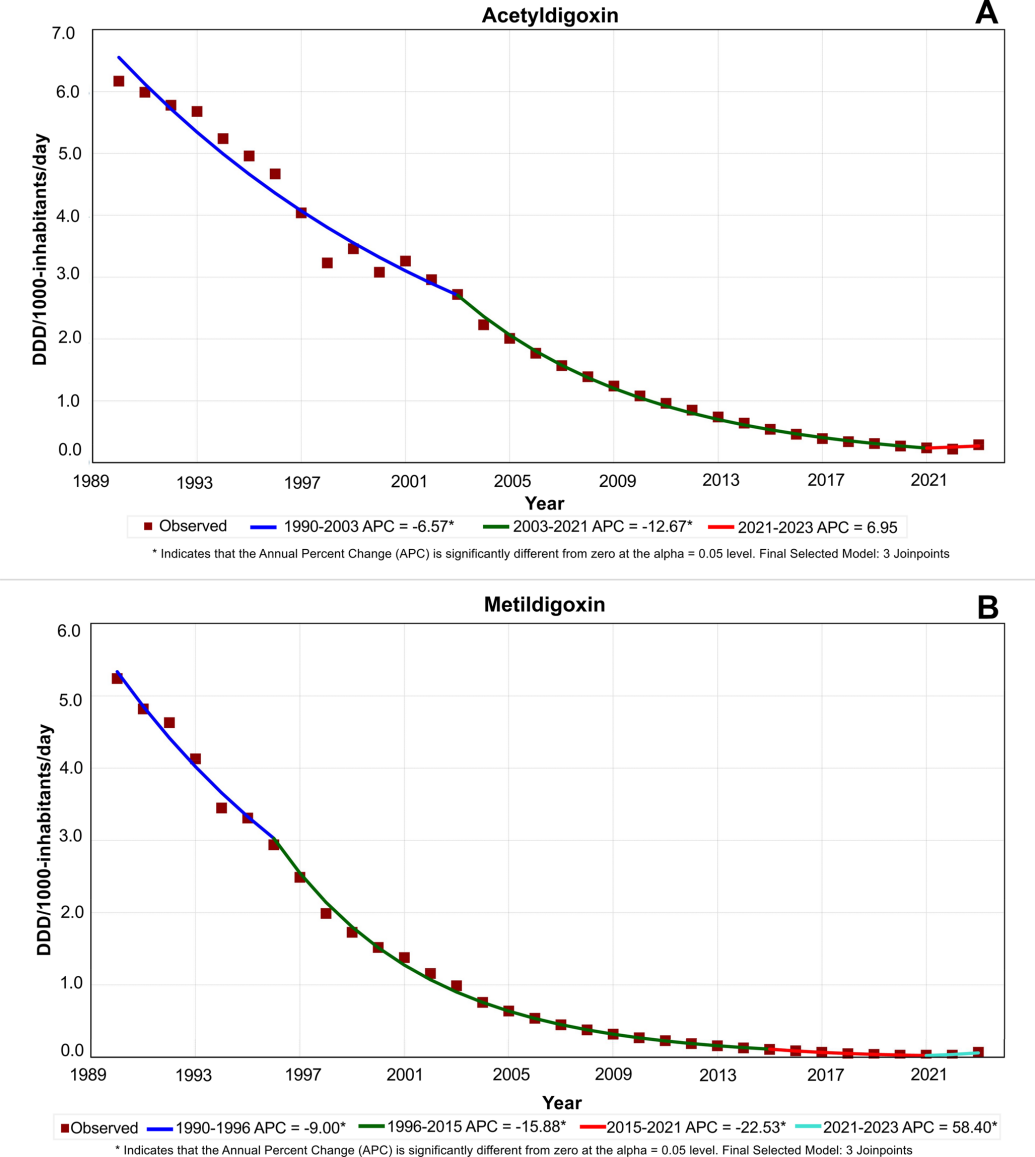
Multi-panel display of joinpoint analyses for digitoxin and digoxin use (DDD/1000-inhabitants/day) from 1990–2023. (**A**) Acetyldigoxin; (**B**) Metildigoxin. Brown square points display the DDD/1000-inhabitants/day. The coloured lines represent the automatically recognized trend segments. A different colour represents a different trend segment. Detailed data tables according the DDD/1000-inhabitants/day for the four NKA-inhibitors (APC, Upper- and lower CI, P-values) are displayed in Tables 2–5 in the supplementary material. Y-axis was adjusted individually for each substance to improve visualization of substance specific trends. Comparison should focus on relative trends

For the investigated timeframe (1990–2023), the DDD of all four NKA-inhibitor substances are continuously decreased. The pre-/post-study analyses of the five major studies DIG (1997), AFFIRM (2002), Rathore et al. (2003), Ahmed et al. (2006) and Castagno et al. (2012) found negative APC values after each study, indicating a decline in DDD after the releasing date of the individual study. Although the pre/post analyses suggest a temporal association, they did not formally test the differences between the periods. The actual changes in DDD-trends were identified by our whole-Joinpoint trend analyses over the complete investigation: For digitoxin the analysis identified significant DDD-trend-changes after 1994 and 2012 (Fig. 4A). Digoxin showed significant DDD-trend-changes after 2006, 2015 and 2021 (Fig. 4B). For acetyldigoxin the analysis identified significant DDD-trend-changes after 2003 and 2021 (Fig. 5A). For Metildigoxin the significant DDD-trend-changes were identified after 1996, 2015 and 2021 (Fig. 5B). The detailed data-results of the whole-joinpoint analyses are displayed in Tables S6-9 in the supplement. Our analysis found no direct association between the study release dates and a subsequent change in DDD-trends. All DDD numbers showed a statistically significant DDD-trend change after 2021: increased DDD-volumes for digoxin, acetyldigoxin and metildigoxin, digitoxin-DDD are decreased.

**Fig. 6:**
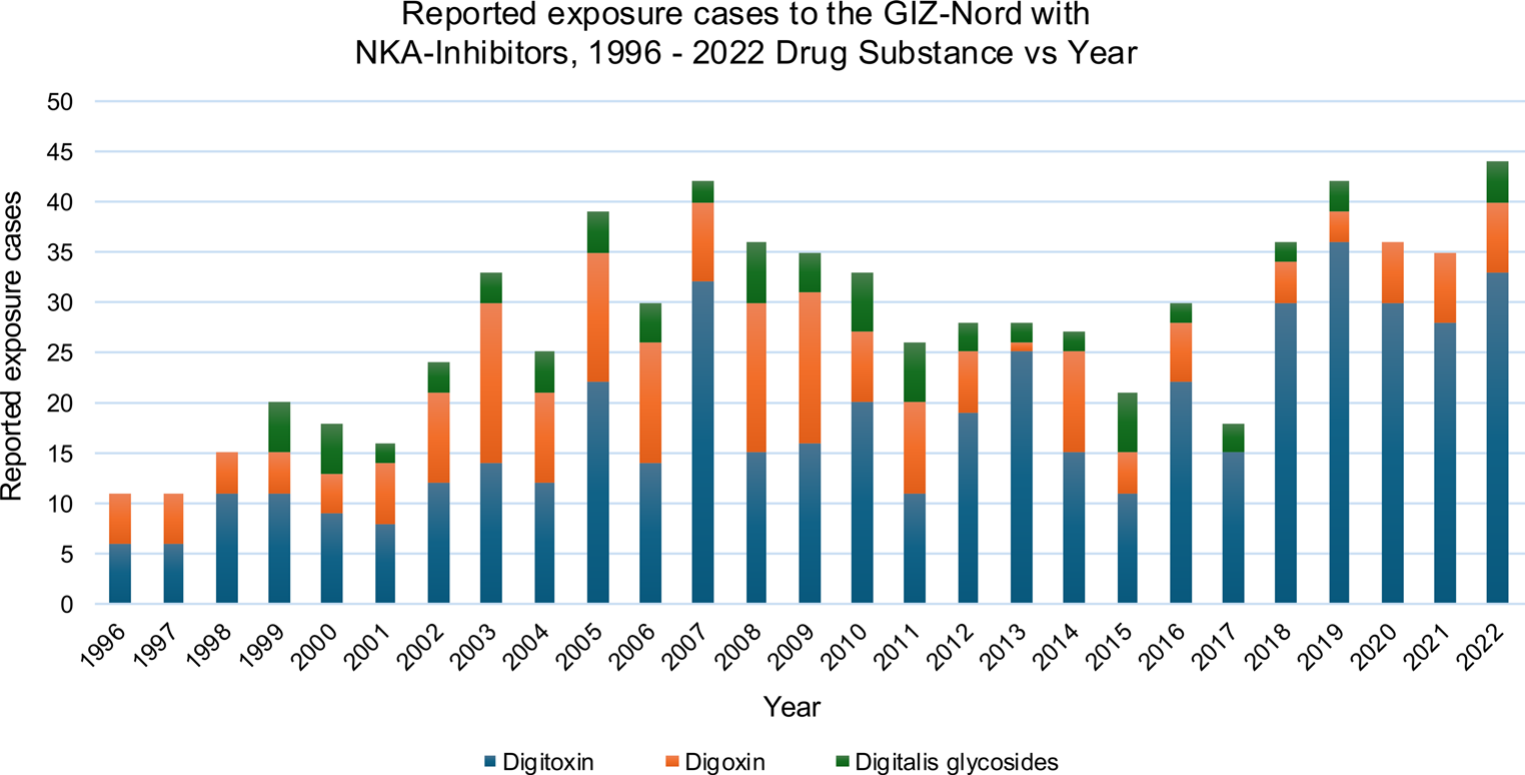
Total annual number of reported exposure cases with NKA-Inhibitors of the GIZ-Nord catchment area presented in a combined column chart: digitoxin (blue), digoxin (orange) and unspecified digitalis glycosides (green). (Data source: GIZ-Nord Poison Centre)

### Poison center-reported exposure cases

**Fig. 7:**
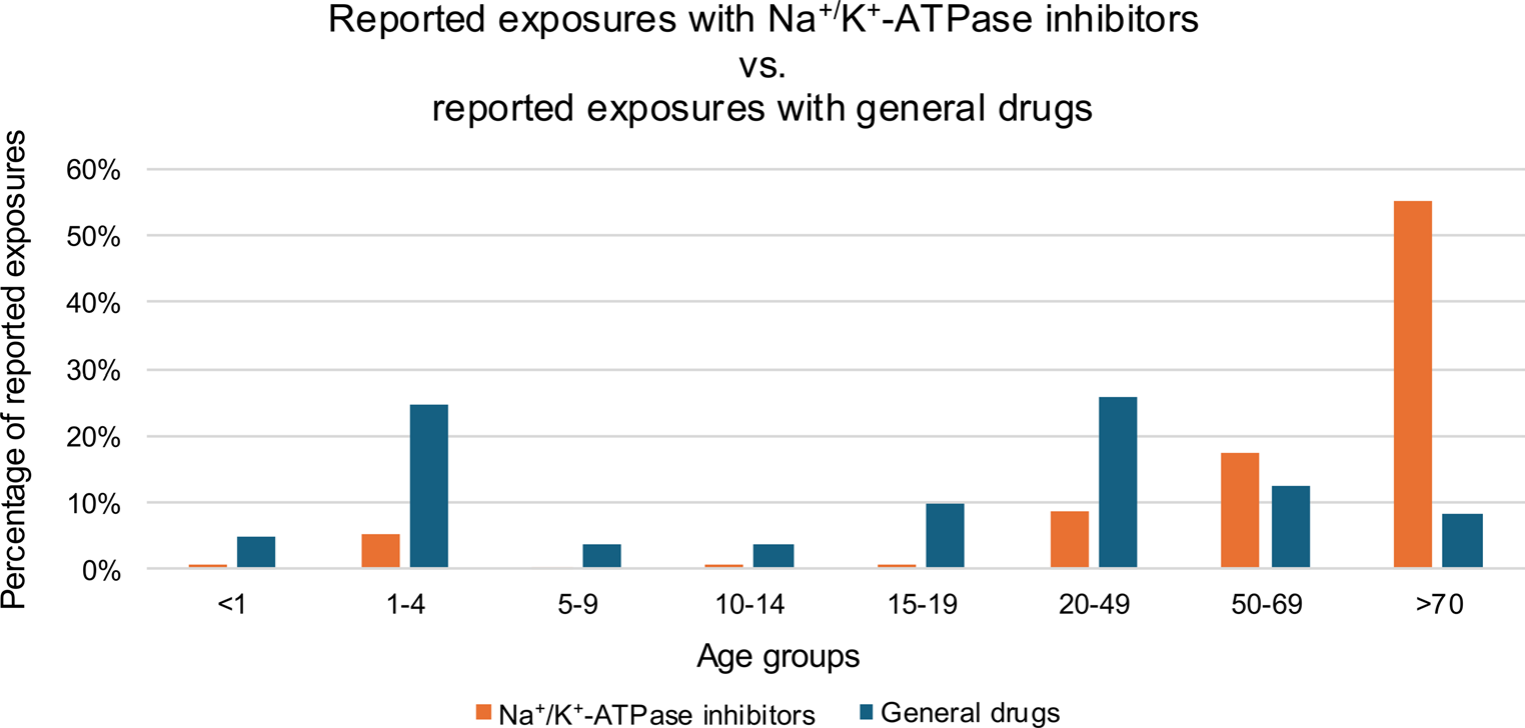
Percentage distribution of reported exposure cases with NKA-inhibitors across the individual age groups presented in a column chart based on data received by the GIZ-Nord: Registered exposure cases with NKA-inhibitors (orange), registered general drug exposure cases (blue) (Data source: GIZ-Nord)

Although the absolute number of exposure cases remains low, reported exposure cases for digitoxin increased from 8 to 10 cases per year to 30–35 cases per year from 1996 to 2022 (Fig. 6), therefore tripling over the investigated timeframe. The reported exposure cases by the GIZ-Nord poisons center show a markedly different age distribution for NKA-inhibitors compared to other reported substances (Fig. 7). Reported cases with NKA-inhibitors are disproportionally more represented in older age groups, especially in patients > 70 years. Reported exposure cases with other substances are more uniformly distributed across age groups.

More than 50% of the reported exposure cases with NKA-inhibitors are found within the age group >70 years, compared to less than 10% in the exposures with other drugs. This highlights the overrepresentation of elderly patients in the reported exposure cases with NKA-inhibitors.

**Fig. 8:**
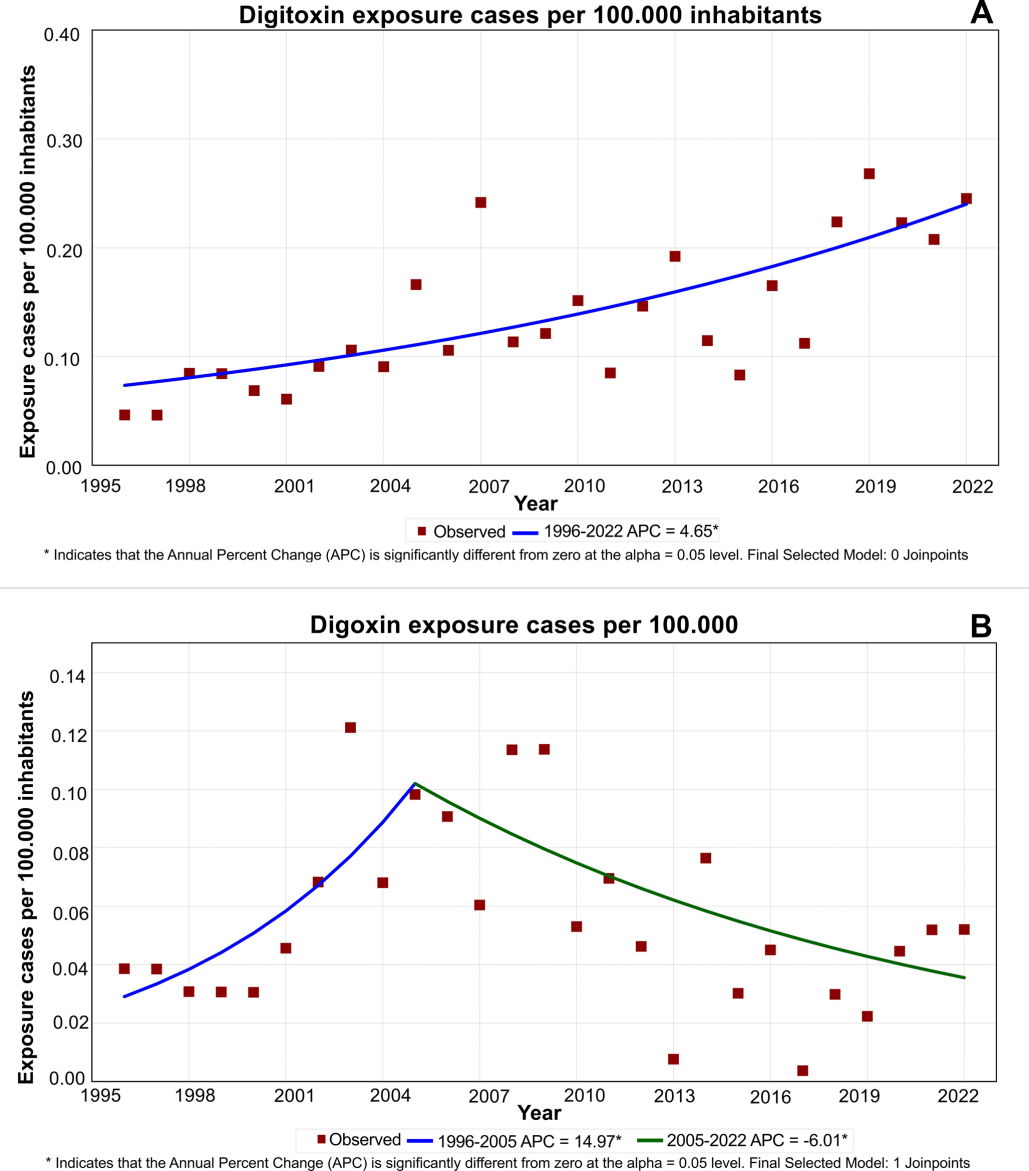
Joinpoint analyses for digitoxin and digoxin related exposure cases from 1996–2022 based on GIZ-Nord data. (**A**) Digitoxin; (**B**) Digoxin. Brown square points display the reported exposure cases per 100.000-inhabitants. The coloured lines represent the automatically recognized trend segments. Detailed results (APC, Upper- and lower CI, P-values) are displayed in Tables 10–13 in the supplementary material. Absolute case numbers shown in Fig. 6. Y-axis was adjusted individually for each substance to improve visualization of substance specific trends.Comparison should focus on relative trends

**Fig. 9:**
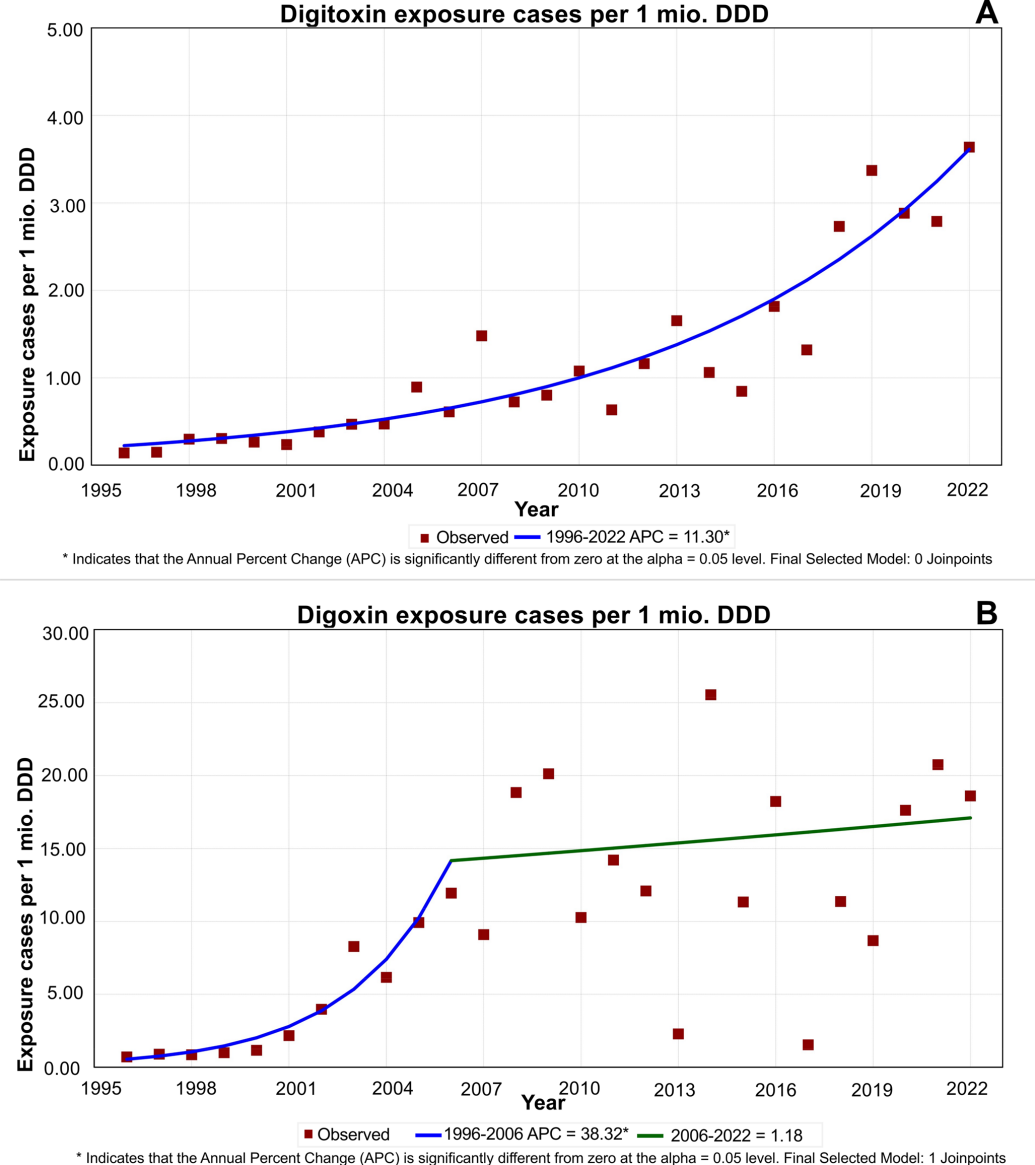
Joinpoint analyses for digitoxin and digoxin related exposure cases per 1 mio. DDD from 1996–2022 based on a model calculation of GIZ-Nord data and exposure-adjusted DDD values. (**A**) Digitoxin; (**B**) Digoxin. Brown square points display the reported exposure cases per 1 mio. DDD. The coloured lines represent the automatically recognized trend segments. Detailed data tables (APC, Upper- and lower CI, P-values) are displayed in Tables 10–13 in the sup–plementary material. Absolute case numbers presented in Fig. 6.Y-axis was adjusted individually for each substance to improve visualization of substance specific trends. Comparison should focus on relative trends

### Joinpoint

#### Joinpoint - Poison center-reported exposure cases

The population adapted joinpoint calculation for reported digitoxin-related exposure cases/100.000 inhabitants shows a significant and continuous increase from 1995 to 2022 (APC = + 4.65%/year, *p* < 0.00001) (Fig. 8A). For digoxin our population adapted analysis showed a strong increase in reported exposure-cases/100.000 inhabitants from 1996 to 2005 (APC = 14.97%, *p* < 0.004). From 2005 to 2022 the reported exposure cases/100.000 inhabitants are decreasing (APC = -6.01%, *p* < 0.000001) (Fig. 8B). The exposure adapted joinpoint analysis per 1mio. digitoxin DDD showed an even more drastic increase (APC = + 11.30%/year, *p* < 0.000001), indicating a substantial increase in reported exposure cases relative to the estimated prescription volume (Fig. 9A). The exposure adapted joinpoint analysis per 1mio. digoxin DDD found a strong and significant increase from 1996 to 2006 (APC = 38.32%, *p* < 0.000001) followed by an insignificant stable trend from 2006 to 2022 and large variabitlity of individual values (APC = 1.18%, *p* = 0.79) (Fig. 9B). The reported exposure cases show noticeable year-to-year variability especially for digoxin, which should be considered for the interpretation of the magnitude of the overall trend. Detailed results are shown in the supplementary material (Tables 10–13).

#### DDD-half time

In the DDD-half-time calculations for NKA-inhibitors, Digitoxin exhibited the highest value whereas Digoxin demonstrated the lowest calculated half-time:

**Table 2:** Results of the half-time-calculations in prison correlated with the study count on ClinicalTrials.gov

	Digitoxin	Digoxin	Acetyldigoxin	Metildigoxin
DDD-half-time (in years)	12.15	3.50	8.34	6.13
Studies (with results)	0	3	0	0

**Fig. 10:**
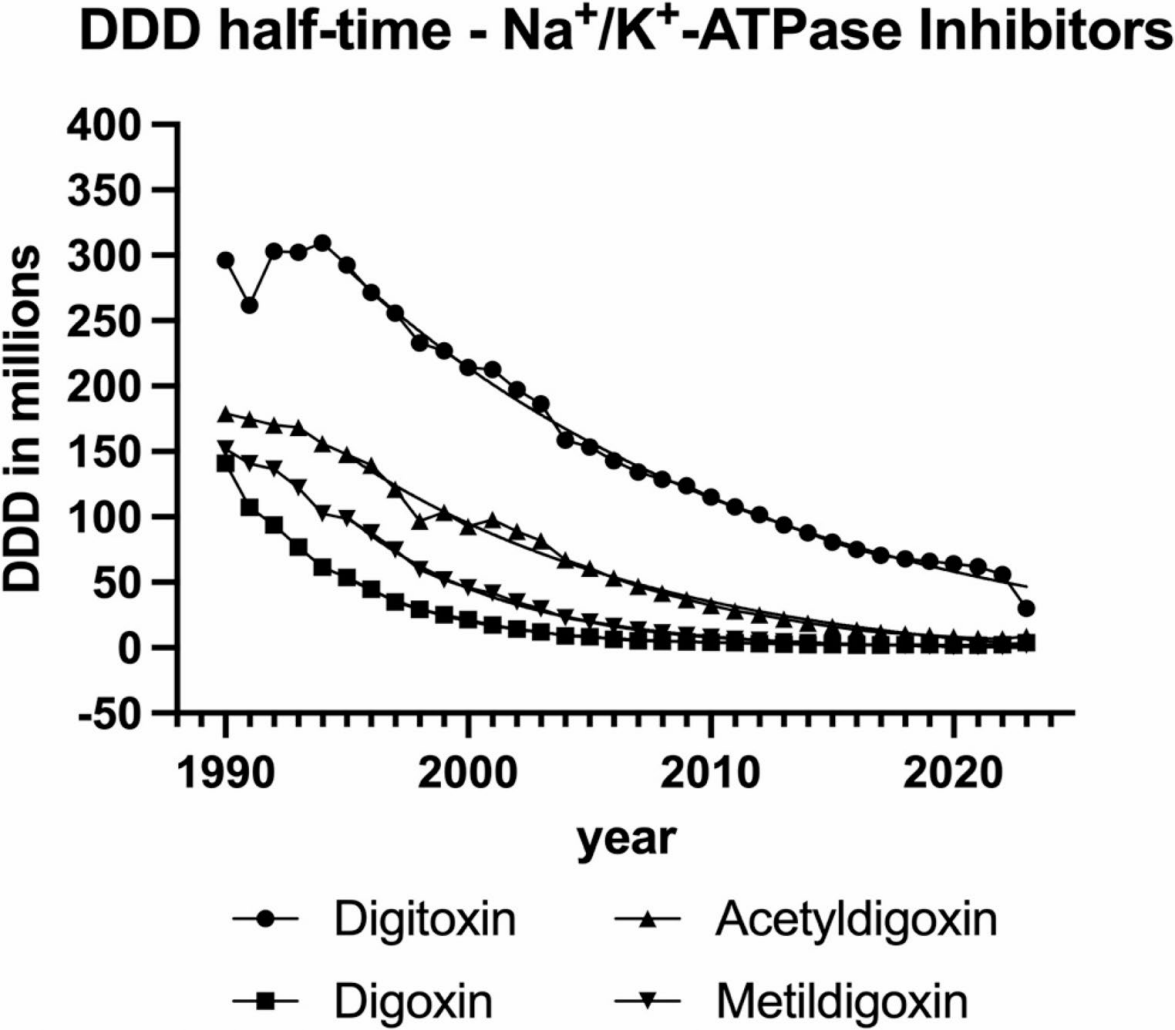
Calculation of the DDD-half-time for the individual NKA-inhibitor substances

With the longest DDD-half-time digitoxin showed the flattest and most steady decline curve, whereas digoxin showed the steepest decline and therefore the shortest DDD-half-time (Fig. 10). Acetyldigoxin and Metildigoxin lie in-between with declines intermediate to digoxin and digitoxin (Fig. 10). 

## Discussion

Throughout the conducted analysis we identified three key developments for NKA-inhibitors: declining DDD-data, a dissociation between scientific evidence and clinical practice and increasing reported exposure cases involving digitoxin over more than two decades within the GIZ-Nord catchment area. The population-adjusted reported digitoxin-related exposure cases (per 100.000 inhabitants) increased by 4.65% per year (Fig. 8A), while the exposure-adjusted reported exposure cases (per 1mio. DDD) increased by 11.30% per year (Fig. 9A). These results show an increase in the number of reported exposure cases relative to both population size and prescription volume. The decline of NKA-inhibitors in general is mainly attributable to the introduction of better alternatives like ACE-inhibitors, β_1_-adrenoceptor-antagonists and MRCA which provided a much greater therapeutic range and provided a causal treatment instead of symptom-control, as proven in large randomized trials [11–14]. In contrast to these drugs, digoxin failed to show a prognostic benefit for patients with HFrEF according to the DIG study [3]. Although the post-hoc analyses were able to find a potential use of digoxin at certain plasma concentrations, these results did not result in a clear evidence-based indication or guideline for the use of NKA-inhibitors [6, 7, 15]. Reported exposure cases for NKA-inhibitors are disproportionally more common in patient groups of higher age, especially in the age group > 70 years (Fig. 7). This shows a distinct overrepresentation of older patients among the reported exposure cases. These results are in line with the known pharmacokinetic properties of NKA-inhibitors like narrow therapeutic window, long elimination half-life and great potential for drug interactions [16]. The predisposition in elderly patients to suffer from intoxications with NKA-inhibitors and therefore the overrepresentation of elderly patients among the reported exposure cases, is most likely due to lower amount of body mass and the enterohepatic circulation: ‘Digitoxin’s enterohepatic circulation of digitoxin complicates dosing and elimination, particularly in the elderly or in patients with digestive irregularities, leading to prolonged half-life and potential intoxication risk even at low doses’ [16]. Electrolyte disbalances, especially hypokalemia due to the use of diuretics like NKCC-inhibitors, NCC-inhibitors or laxatives like polyethylene glycol can amplify the toxicity of NKA-Inhibitors [16]. The recent discontinuation of Merck’s digitoxin drug production due to economic reasons in 2023 caused the transition of many patients to the use of digoxin [17]. The German Society of Cardiology warns of accumulation tendencies in their statement and advises close monitoring of the digoxin drug levels to avoid intoxication [18]. Based on these developments, prescribing behaviour is not only influenced by clinical evidence but also external factors like market availability [18, 19]. Apart from our toxicological analysis, we observed clear discrepancies between scientific evidence and prescribing behavior. As shown in our DDD-joinpoint-analyses, significant changes in DDD-trends occur mostly detached from the publications of major NKA-inhibitor studies. The most important study for digoxin, the DIG (1997) showed no prognostic benefit for the use of digoxin in HFrEF patients, still no clear trend change in DDD was observed after the publication (Fig. 2A). Instead, the DDD of all four investigated substances declined continuously over time, the detected joinpoints occurred at different years, which suggests the changes might have been caused by better alternatives rather than new scientific evidence on digoxin. The common detected DDD-trend change after 2021 is most probably a result of the digitoxin discontinuation of Merck – more patients had to receive digoxin, acetyldigoxin or metildigoxin as alternative [19]. Therefore, we observed a slight increase in the DDD of the said NKA-inhibitors (Fig. 2B). These discrepancies were further quantified by the conducted DDD-half-time analysis: Digitoxin showed the longest DDD-half-time (12.45 years) of all four substances and therefore a particularly slow decline of its clinical use, despite the missing clinical evidence until DIGIT-HF in 2025 (Table 2, Fig. 10). On the contrary, digoxin showed a short DDD-half-time (3.50 years), this seems more in line with the stronger but still limited evidence base (Table 2, Fig. 10). Acetyldigoxin and metildigoxin lie in between with DDD-half-times of 8.34 years and 6.13 years, despite the unavailability of clinical trials to investigate the primary efficacy of these drugs (Table 2, Fig. 10). These findings put together implicate that market structure, historical use and pharmacokinetic convenience are stronger influencing factors for prescribing NKA-inhibitors than the availability of scientific evidence. Recently, the results of the DIGIT-HF study have reignited the discussion and the scientific interest in NKA-inhibitors. For the first time there seems to be scientific proof for selected patient groups with HFrEF in accordance with a guideline-based HF-therapy [5]. The results of the DIGIT-HF study therefore constitute a great improvement in the evaluation of the efficacy of NKA-inhibitors but do not address long-term drug safety. Our analysis based on poison center data, shows reported exposure cases under real world conditions within the GIZ-Nord catchment area. Under current prescribing conditions, the re-introduction of digitoxin as add-on drug for HF may provide a new increase in reported exposure cases. From a toxicological perspective the recent rise in reported cases speaks against an uncritical re-introduction of NKA-inhibitors into HF-therapy. Strict therapeutic drug level monitoring may be particularly important, especially for elderly patients.

## Limitations

Certain limitations must be considered.

The poison-center data displayed were available only from GIZ-Nord which covers the 4 northern federal German States of Bremen, Hamburg, Niedersachsen and Schleswig-Holstein. Despite several inquiries to other German poison information centers, we were’nt able to get comparable datasets. The data from the GIZ-Nord display only reported exposure rates, the actual numbers may be higher. Also, the GIZ-Nord data represents reported exposure cases rather than confirmed clinical intoxication cases. The cases may include multi-agent exposures, but only cases in which NKA-inhibitors were assessed as the primary agent were included. Poison center data are prone to reporting bias and may be influenced by physician reporting, awareness or patient healthcare-seeking. The actual exposure cases may be under- or overestimated. Although the WidO DDD datasets provide a reliable, validated source, it can’t be broken down by patient demographics, individual indications or specialty. Therefore, the interpretation of DDD-data is bound to Germanys general prescription of NKA-inhibitors and does not refer to specific subpopulations or special clinical contexts. Also, the joinpoint regression program required at least four data points after a potential joinpoint for valid trend assessment, therefore events after 2018 could not be tested. This can lead to underestimation of recent trend changes. Even though we performed a thorough literature review this study intentionally provides only targeted contextualization of major studies in terms of pharmacological, pharmacoeconomic and toxicological findings to support interpretation of the received data. It does not constitute a full systematic review. Our focus was restricted to the most relevant RCTs and the major publications within the investigated timeframe. For this reason, smaller or unpublished studies may not have been covered by our research. Finally, the performed DDD-half-time calculation model used to link evidence and prescription trends assumes direct temporal association between the publication of scientific studies and changes in clinical practice. These calculations cannot display real-world influences like physicians’ individual prescription behavior, cost pressure, delays in the development of new guidelines or market factors. Despite of these limitations, this analysis provides a unique perspective on the pharmacological, pharmacoeconomic and toxicological development of NKA-inhibitors in Germany and offers valuable insights into the scientific foundations of current prescription trends.

## Conclusion

Due to the availability of better alternatives with lager therapeutic windows for the cardiac indications of NKA-inhibitors, the DDD are declining continuously.

Despite the declining prescriptions of NKA-inhibitors, reported exposure cases are increasing for digitoxin and are especially affecting elderly patients. Our DDD-joinpoint-analyses and DDD-half-time calculations show a clear discrepancy between scientific evidence and clinical-practice. Digitoxin had the highest prescription volumes on the German market even though there was no RCT prior to DIGIT-HF investigating its efficacy as primary drug. Although the DIGIT-HF study provided evidence for a beneficial use of NKA-inhibitors in patients with HFrEF, our data demonstrates the importance of a strict and careful monitoring and selection of patients receiving NKA-inhibitors. The indication especially for the future use of digitoxin should be established carefully after the evaluation for potential drug-interactions, kidney and electrolyte status and always be accompanied with serum-level monitoring. For an in-depth evaluation of current and future use of drugs with narrow therapeutic windows like NKA-inhibitors, toxicological real-world data should be included to sufficiently and preventatively protect vulnerable patient groups.

## Supplementary Information

Below is the link to the electronic supplementary material.

Supplementary Material 1 (DOCX 71.7 KB)

## Data Availability

All source data of this study are available upon reasonable request.

## Abbreviations

ACE-Inhibitors: Angiotensin-converting-enzyme inhibitors
BfArM: Bundesinstitut für Arzneimittel und Medizinprodukte (Federal Institute for Drugs and Medical Devices)
CCB: Calcium channel blocker
DDD: Defined daily dose
GIZ-Nord: Giftinformationszentrum-Nord (GIZ-Nord Poisons Centre)
HF: Heart failure
HFrEF: Heart failure with reduced ejection fraction
NKA-inhibitors: Na^+^/K^+^-ATPase inhibitors
RCT: Randomized controlled trial
SDC: Serum digoxin concentration
WidO: Wissenschaftliches Institut der AOK (Scientific Institute of the AOK)
