# Supplementary material for: Critical analysis of digitalis glycosides: declining use but increasing poison-center exposure cases for digitoxin
Source: Eur J Clin Pharmacol. 2026 Jun 23;82(7):186. doi: 10.1007/s00228-026-04099-3 (PMC13290837; doi:10.1007/s00228-026-04099-3)
Supplement: Supplementary file 1 — Supplementary Material 1 (DOCX 71.7 KB) [file 228_2026_4099_MOESM1_ESM.docx]

## **Supplemental data**

## **Critical analysis of cardiac glycosides: declining use but increasing poison-center exposure cases for digitoxin**

## **Paul Antek Matthias Ecker^1^, Andreas Schaper^2^,**

## **Hans Jörg Bräunig^2^, Roland Seifert^1^**

## **^1^Institute of Pharmacology Hannover Medical School D-30625 Hannover, Germany**

## **^2^GIZ-Nord Poisons Centre for Bremen, Hamburg, Niedersachsen and Schleswig-Holstein Georg-August-University of Göttingen D-37075 Göttingen, Germany**

| **SECTION** | **ITEM** | **PRISMA-ScR CHECKLIST ITEM** | **REPORTED ON PAGE #** |
| --- | --- | --- | --- |
| **TITLE** | | | |
| Title | 1  **Table S1** Prisma-ScR Checklist | Identify the report as a scoping review. | 1 |
| **ABSTRACT** | | | |
| Structured summary | 2 | Provide a structured summary that includes (as applicable): background, objectives, eligibility criteria, sources of evidence, charting methods, results, and conclusions that relate to the review questions and objectives. | 2-3 |
| **INTRODUCTION** | | | |
| Rationale | 3 | Describe the rationale for the review in the context of what is already known. Explain why the review questions/objectives lend themselves to a scoping review approach. | 3-4 |
| Objectives | 4 | Provide an explicit statement of the questions and objectives being addressed with reference to their key elements (e.g., population or participants, concepts, and context) or other relevant key elements used to conceptualize the review questions and/or objectives. | 4 |
| **METHODS** | | | |
| Protocol and registration | 5 | Indicate whether a review protocol exists; state if and where it can be accessed (e.g., a Web address); and if available, provide registration information, including the registration number. | No protocol registered |
| Eligibility criteria | 6 | Specify characteristics of the sources of evidence used as eligibility criteria (e.g., years considered, language, and publication status), and provide a rationale. | 5-6 |
| Information sources* | 7 | Describe all information sources in the search (e.g., databases with dates of coverage and contact with authors to identify additional sources), as well as the date the most recent search was executed. | 4-5 |
| Search | 8 | Present the full electronic search strategy for at least 1 database, including any limits used, such that it could be repeated. | 5-6 |
| Selection of sources of evidence† | 9 | State the process for selecting sources of evidence (i.e., screening and eligibility) included in the scoping review. | 5 |
| Data charting process‡ | 10 | Describe the methods of charting data from the included sources of evidence (e.g., calibrated forms or forms that have been tested by the team before their use, and whether data charting was done independently or in duplicate) and any processes for obtaining and confirming data from investigators. | 5-6 |
| Data items | 11 | List and define all variables for which data were sought and any assumptions and simplifications made. | 5-6; 11 |
| Critical appraisal of individual sources of evidence§ | 12 | If done, provide a rationale for conducting a critical appraisal of included sources of evidence; describe the methods used and how this information was used in any data synthesis (if appropriate). | 6 |
| Synthesis of results | 13 | Describe the methods of handling and summarizing the data that were charted. | 6-7 |
| **RESULTS** | | | |
| Selection of sources of evidence | 14 | Give numbers of sources of evidence screened, assessed for eligibility, and included in the review, with reasons for exclusions at each stage, ideally using a flow diagram. | 8-12 |
| Characteristics of sources of evidence | 15 | For each source of evidence, present characteristics for which data were charted and provide the citations. | 11 |
| Critical appraisal within sources of evidence | 16 | If done, present data on critical appraisal of included sources of evidence (see item 12). | 11 |
| Results of individual sources of evidence | 17 | For each included source of evidence, present the relevant data that were charted that relate to the review questions and objectives. | 8-9 |
| Synthesis of results | 18 | Summarize and/or present the charting results as they relate to the review questions and objectives. | 9-17 |
| **DISCUSSION** | | | |
| Summary of evidence | 19 | Summarize the main results (including an overview of concepts, themes, and types of evidence available), link to the review questions and objectives, and consider the relevance to key groups. | 17-19 |
| Limitations | 20 | Discuss the limitations of the scoping review process. | 20-21 |
| Conclusions | 21 | Provide a general interpretation of the results with respect to the review questions and objectives, as well as potential implications and/or next steps. | 21-22 |
| **FUNDING** | | | |
|  | 22 | Describe sources of funding for the included sources of evidence, as well as sources of funding for the scoping review. Describe the role of the funders of the scoping review. | 22 |

**Joinpoint analysis – pre-/post study analyses**

**Digitoxin**

| Event name | Event year | APC-Pre(%) | 95% CI Pre (Lower) | 95% CI Pre (Upper) | APC Post (%) | 95% Post (Lower) | 95% CI Post (Upper) | CI Overlap (yes/no) | Trend changed (yes/no) |
| --- | --- | --- | --- | --- | --- | --- | --- | --- | --- |
| DIG Trial | 1997 | -1.55 | -3.81 | 0.84 | -6.48 | -7.30 | -5.67 | No | yes |
| Affirm et al. | 2002 | -3.74 | -4.99 | -2.48 | -6.63 | -7.86 | -5.43 | No | Yes |
| Rathore et al. | 2003 | -3.92 | -5.06 | -2.78 | -6.61 | -7.93 | -5.28 | No | Yes |
| Ahmed et al. | 2006 | -4.80 | -5.67 | -3.93 | -6.8 | -8.67 | -5.25 | No | Yes |
| Castagno et al. | 2012 | -5.27 | -5.81 | -4.73 | -7.93 | -11.51 | -4.22 | No | Yes |

***Table S2****Results of the individual pre-/post-study joinpoint analysis based on digitoxin utilization (DDD/1000-inhabitants/day). APC and 95% CI were calculated by using joinpoint regression with 0 joinpoints for the individual segments. Non-overlapping CI’s indicate a statistically significant change in APC.*

**Digoxin**

| Event name | Event year | APC-Pre(%) | 95% CI Pre (Lower) | 95% CI Pre (Upper) | APC Post (%) | 95% Post (Lower) | 95% CI Post (Upper) | CI Overlap (yes/no) | Trend changed (yes/no) |
| --- | --- | --- | --- | --- | --- | --- | --- | --- | --- |
| DIG Trial | 1997 | -17.77 | -19.10 | -16.40 | -9.92 | -12.05 | -7.78 | No | Yes |
| Affirm et al. | 2002 | -17.27 | -17.64 | -16.90 | -7.26 | -9.65 | -4.80 | No | Yes |
| Rathore et al. | 2003 | -17.22 | -17.57 | -16.86 | -6.57 | -9.06 | -4.02 | No | Yes |
| Ahmed et al. | 2006 | -17.41 | -17.82 | -16.99 | -4.71 | -8.26 | -0.88 | No | Yes |
| Castagno et al. | 2012 | -16.29 | -16.94 | -15.65 | 1.69 | -1.94 | 5.51 | Yes | Yes |

***Table S3,*** *Results of the individual pre-/post-study joinpoint analysis based on digoxin utilization (DDD/1000-inhabitants/day). APC and 95% CI were calculated by using joinpoint regression with 0 joinpoints for the individual segments. Non-overlapping CI’s indicate a statistically significant change in APC.*

**Acetyldigoxin**

| Event name | Event year | APC-Pre(%) | 95% CI Pre (Lower) | 95% CI Pre (Upper) | APC Post (%) | 95% Post (Lower) | 95% CI Post (Upper) | CI Overlap (yes/no) | Trend changed (yes/no) |
| --- | --- | --- | --- | --- | --- | --- | --- | --- | --- |
| DIG Trial | 1997 | -5.50 | -6.69 | -4.25 | -11.41 | -12.03 | -10.80 | No | yes |
| Affirm et al. | 2002 | -6.70 | -8.13 | -5.25 | -12.04 | -12.72 | -11.33 | No | yes |
| Rathore et al. | 2003 | -6.66 | -7.73 | -5.58 | -11.98 | -12.73 | -11.24 | No | yes |
| Ahmed et al. | 2006 | -7.44 | -8.55 | -6.29 | -11.98 | -13.03 | -10.91 | No | yes |
| Castagno et al. | 2012 | -8.77 | -9.49 | -8.04 | -10.89 | -13.14 | -8.50 | Yes | yes |

***Table S4,*** *Results of the individual pre-/post-study joinpoint analysis based on acetyldigoxin utilization (DDD/1000-inhabitants/day). APC and 95% CI were calculated by using joinpoint regression with 0 joinpoints for the individual segments.*

| Event name | Event year | APC-Pre(%) | 95% CI Pre (Lower) | 95% CI Pre (Upper) | APC Post (%) | 95% Post (Lower) | 95% CI Post (Upper) | CI Overlap (yes/no) | Trend changed (yes/no) |
| --- | --- | --- | --- | --- | --- | --- | --- | --- | --- |
| DIG Trial | 1997 | -10.02 | -12.47 | -7.53 | -16.17 | -17.27 | -15.02 | No | yes |
| Affirm et al. | 2002 | -12.23 | -13.38 | -11.05 | -16.21 | -17.91 | -14.43 | No | yes |
| Rathore et al. | 2003 | -12.42 | -13.46 | -11.38 | -16.09 | -17.98 | -14.15 | No | yes |
| Ahmed et al. | 2006 | -13.46 | -14.44 | -12.44 | -15.95 | -18.47 | -13.22 | No | yes |
| Castagno et al. | 2012 | -14.50 | -15.20 | -13.80 | -14.05 | -21.42 | -5.80 | Yes | yes |

**Metildigoxin**

***Table S5,*** *Results of the individual pre-/post-study joinpoint analysis based on metildigoxin utilization (DDD/1000-inhabitants/day). APC and 95% CI were calculated by using joinpoint regression with 0 joinpoints for the individual segments.*

| **Estimated Joinpoints** | | | | | | | |
| --- | --- | --- | --- | --- | --- | --- | --- |
| **Joinpoint** | **Estimate** | **Lower Cl** | **Upper Cl** |  |  |  |  |
| 1 | 1994 | 1992 | 1998 |  |  |  |  |
| 2 | 2021 | 2020 | 2021 |  |  |  |  |
| **Annual Percent Change (APC)** | | | | | | | |
| Segment | Lower Endpoint | Upper Endpoint | APC | Lower Cl | Upper CI | Test Statistic | P-Value |
| 1 | 1990 | 1994 | 1.4801 | -3.0458 | 10.1503 | -- | 0.425515 |
| 2 | 1994 | 2021 | -5.9755* | -6.3056 | -5.6958 | -- | 0.000400 |
| 3 | 2021 | 2023 | -24.9782* | -29.2191 | -16.3792 | -- | < 0.000001 |
| * Indicates that the Annual Percent Change (APC) is significantly different from zero at the alpha = 0.05 level. | | | | | | | |

***Table S6,*** *Data trends for the whole-joinpoint analysis of digitoxin – tabular presentation of the number and range of the individual identified joinpoints and the annual perecent change for the individual segments between the identified joinpoints.*

| **Estimated Joinpoints** | | | | | | | |
| --- | --- | --- | --- | --- | --- | --- | --- |
| Joinpoint | Estimate | Lower CI | Upper CI |  |  |  |  |
| 1 | 2006 | 2004 | 2007 |  |  |  |  |
| 2 | 2015 | 2014 | 2016 |  |  |  |  |
| 3 | 2021 | 2020 | 2021 |  |  |  |  |
| **Annual Percent Change (APC)** | | | | | | | |
| Segment | Lower Endpoint | Upper Endpoint | APC | Lower Cl | Upper Cl | Test Statistic | P-Value |
| 1 | 1990 | 2006 | -17.4578* | -17.9481 | -17.0305 | -- | < 0,000001 |
| 2 | 2006 | 2015 | -11.5418* | -12.9999 | -10.0333 | -- | < 0,000001 |
| 3 | 2015 | 2021 | -0.9251 | -3.4141 | 2.0523 | -- | 0,368326 |
| 4 | 2021 | 2023 | 34.2493* | 23.5890 | 41.9476 | -- | < 0,000001 |
| * Indicates that the Annual Percent Change (APC) is significantly different from zero at the alpha = 0.05 level. | | | | | | | |

***Table S7,*** *Data trends for the whole-joinpoint analysis of digoxin – tabular presentation of the number and range of the individual identified joinpoints and the annual perecent change for the individual segments between the identified joinpoints.*

| **Estimated Joinpoints** | | | | | | | |
| --- | --- | --- | --- | --- | --- | --- | --- |
| Joinpoint | Estimate | Lower Cl | Upper CI |  |  |  |  |
| 1 | 2003 | 2002 | 2005 |  |  |  |  |
| 2 | 2021 | 2019 | 2021 |  |  |  |  |
| **Annual Percent Change (APC)** | | | | | | | |
| Segment | Lower  Endpoint | Upper  Endpoint | APC | Lower Cl | Upper CI | Test Statistic | P-Value |
| 1 | 1990 | 2003 | -6.5719* | -7.4111 | -5.5771 | -- | < 0.000001 |
| 2 | 2003 | 2021 | -12.6685* | -13.3946 | -12.2064 | -- | < 0.000001 |
| 3 | 2021 | 2023 | 6.9497 | -5.4517 | 12.9366 | -- | 0.245551 |
| * Indicates that the Annual Percent Change (APC) is significantly different from zero at the alpha = 0.05 level. | | | | | | | |

***Table S8,*** *Data trends for the whole-joinpoint analysis of acetyldigoxin – tabular presentation of the number and range of the individual identified joinpoints and the annual perecent change for the individual segments between the identified joinpoints.*

| **Estimated Joinpoints** | | | | | | | |
| --- | --- | --- | --- | --- | --- | --- | --- |
| Joinpoint | Estimate | Lower CI | Upper CI |  |  |  |  |
| 1 | 1996 | 1993 | 2000 |  |  |  |  |
| 2 | 2015 | 2012 | 2017 |  |  |  |  |
| 3 | 2021 | 2021 | 2021 |  |  |  |  |
| **Annual Percent Change (APC)** | | | | | | | |
| Segment | Lower  Endpoint | Upper  Endpoint | APC | Lower Cl | Upper CI | Test Statistic | P-Value |
| 1 | 1990 | 1996 | -9.0000* | -12.1365 | -1.0926 | -- | 0,038792 |
| 2 | 1996 | 2015 | -15.8760* | -16.5947 | -15.1888 | -- | < 0,000001 |
| 3 | 2015 | 2021 | -22.5346* | -28.8427 | -20.0099 | -- | < 0,000001 |
| 4 | 2021 | 2023 | 58.3955* | 36.1188 | 77.5622 | -- | < 0,000001 |
| * Indicates that the Annual Percent Change (APC) is significantly different from zero at the alpha = 0.05 level. | | | | | | | |

***Table S9,*** *Data trends for the whole-joinpoint analysis of metildigoxin – tabular presentation of the number and range of the individual identified joinpoints and the annual perecent change for the individual segments between the identified joinpoints.*

**GIZ-Nord data analysis with joinpoint:**

| **Annual Percent Change (APC)** | | | | | | | |
| --- | --- | --- | --- | --- | --- | --- | --- |
| Segment | Lower Endpoint | Upper Endpoint | APC | Lower CI | Upper CI | Test Statistic | P-Value |
| 1 | 1996 | 2022 | 4.6545* | 3.2106 | 6.6171 | -- | <0.000001 |
| * Indicates that the Annual Percent Change (APC) is significantly different from zero at the alpha = 0.05 level. | | | | | | | |

***Table S10,*** *Data trends for the joinpoint analysis for digitoxin related exposure cases cases per 100.000 inhabitants – tabular presentation of the annual perecent change for the identified trend segment.*

| **Annual Percent Change (APC)** | | | | | | | |
| --- | --- | --- | --- | --- | --- | --- | --- |
| Segment | Lower Endpoint | Upper Endpoint | APC | Lower CI | Upper CI | Test Statistic | P-Value |
| 1 | 1996 | 2022 | 11.8178* | 10.0082 | 13.6302 | -- | <0.000001 |
| * Indicates that the Annual Percent Change (APC) is significantly different from zero at the alpha = 0.05 level. | | | | | | | |

***Table S11,*** *Data trends for the joinpoint analysis of digitoxin related exposure cases per 1 mio. DDD from 1996-2022 based on a model calculation of GIZ-north data and adapted DDD values. DDD values were adapted percentagewise based on Germanys whole population and the GIZ-Nord population share. – tabular presentation of the annual perecent change for the identified trend segment.*

| **Estimated Joinpoints** | | | | | | | |
| --- | --- | --- | --- | --- | --- | --- | --- |
| Joinpoint | Estimate | Lower CI | Upper CI |  |  |  |  |
| 1 | 2005 | 2001 | 2010 |  |  |  |  |
| **Annual Percent Change (APC)** | | | | | | | |
| Segment | Lower  Endpoint | Upper  Endpoint | APC | Lower Cl | Upper CI | Test Statistic | P-Value |
| 1 | 1996 | 2005 | 14.9672* | 5.5955 | 52.7911 | -- | 0.000400 |
| 2 | 2005 | 2022 | -6.0079* | -12.3908 | -2.9101 | -- | < 0.000001 |
| * Indicates that the Annual Percent Change (APC) is significantly different from zero at the alpha = 0.05 level. | | | | | | | |

***Table S12,*** *Data trends for the joinpoint analysis of digoxin related exposure cases per 100.000 inhabitants – tabular presentation of the annual perecent change for the identified trend segment.*

| **Estimated Joinpoints** | | | | | | | |
| --- | --- | --- | --- | --- | --- | --- | --- |
| Joinpoint | Estimate | Lower CI | Upper CI |  |  |  |  |
| 1 | 2006 | 2004 | 2008 |  |  |  |  |
| **Annual Percent Change (APC)** | | | | | | | |
| Segment | Lower  Endpoint | Upper  Endpoint | APC | Lower Cl | Upper CI | Test Statistic | P-Value |
| 1 | 1996 | 2006 | 38.3164* | 28.2529 | 59,3146 | -- | <0.000001 |
| 2 | 2006 | 2022 | 1.1819 | -4.6248 | 4.8601 | -- | 0.785843 |
| * Indicates that the Annual Percent Change (APC) is significantly different from zero at the alpha = 0.05 level. | | | | | | | |

***Table S13,*** *Data trends for the joinpoint analysis of digoxin related exposure cases per 1 mio. DDD from 1996-2022 based on a model calculation of GIZ-north data and adapted DDD values. DDD values were adapted percentagewise based on Germanys whole population and the GIZ-Nord population share. – tabular presentation of the annual perecent change for the identified trend segment.*

**Sensitivity Analysis for GIZ Nord data - Poisson Variance**

| **Annual Percent Change (APC)** | | | | | | | |
| --- | --- | --- | --- | --- | --- | --- | --- |
| Segment | Lower Endpoint | Upper Endpoint | APC | Lower CI | Upper CI | Test Statistic | P-Value |
| 1 | 1996 | 2022 | 4.6545* | 3,2106 | 6,6171 | -- | <0.000001 |
| * Indicates that the Annual Percent Change (APC) is significantly different from zero at the alpha = 0.05 level. | | | | | | | |

***Figure S14*** *Data trends for the joinpoint analysis of digitoxin related exposure cases per 100.000 inhabitants – tabular presentation of the annual perecent change for the identified trend segment.*

| **Annual Percent Change (APC)** | | | | | | | |
| --- | --- | --- | --- | --- | --- | --- | --- |
| Segment | Lower Endpoint | Upper Endpoint | APC | Lower CI | Upper CI | Test Statistic | P-Value |
| 1 | 1996 | 2022 | 11.3003* | 9.7728 | 13.4050 | -- | <0.000001 |
| * Indicates that the Annual Percent Change (APC) is significantly different from zero at the alpha = 0.05 level. | | | | | | | |

***Figure S15*** *Data trends for the joinpoint analysis of digitoxin related exposure cases per 1 mio. DDD from 1996-2022 based on a model calculation of GIZ-north data and adapted DDD values. DDD values were adapted percentagewise based on Germanys whole population and the GIZ-Nord population share. – tabular presentation of the annual perecent change for the identified trend segment.*

| **Estimated Joinpoints** | | | | | | | |
| --- | --- | --- | --- | --- | --- | --- | --- |
| Joinpoint | Estimate | Lower CI | Upper CI |  |  |  |  |
| 1 | 2005 | 2001 | 2010 |  |  |  |  |
| **Annual Percent Change (APC)** | | | | | | | |
| Segment | Lower  Endpoint | Upper  Endpoint | APC | Lower Cl | Upper CI | Test Statistic | P-Value |
| 1 | 1996 | 2005 | 14.9672* | 5.5955 | 52.7911 | -- | 0.000400 |
| 2 | 2005 | 2022 | -6.0079* | -12.3908 | -2.9101 | -- | < 0.000001 |
| * Indicates that the Annual Percent Change (APC) is significantly different from zero at the alpha = 0.05 level. | | | | | | | |

***Figure S16*** *Data trends for the joinpoint analysis of digoxin related exposure cases per 100.000 inhabitants – tabular presentation of the annual perecent change for the identified trend segment.*

| **Estimated Joinpoints** | | | | | | | |
| --- | --- | --- | --- | --- | --- | --- | --- |
| Joinpoint | Estimate | Lower CI | Upper CI |  |  |  |  |
| 1 | 2006 | 2004 | 2008 |  |  |  |  |
| **Annual Percent Change (APC)** | | | | | | | |
| Segment | Lower  Endpoint | Upper  Endpoint | APC | Lower Cl | Upper CI | Test Statistic | P-Value |
| 1 | 1996 | 2006 | 38.3164* | 28.2529 | 59,3146 | -- | <0.000001 |
| 2 | 2006 | 2022 | 1.1819 | -4.6248 | 4.8601 | -- | 0.785843 |
| * Indicates that the Annual Percent Change (APC) is significantly different from zero at the alpha = 0.05 level. | | | | | | | |

***Figure S17*** *Data trends for the joinpoint analysis of digoxin related exposure cases per 1 mio. DDD from 1996-2022 based on a model calculation of GIZ-north data and adapted DDD values. DDD values were adapted percentagewise based on Germanys whole population and the GIZ-Nord population share. – tabular presentation of the annual perecent change for the identified trend segment.*
